# Health-Related Predictors of Changes in Cognitive Status in Community-Dwelling Older Individuals

**DOI:** 10.3389/fnagi.2022.876359

**Published:** 2022-06-20

**Authors:** Caterina Trevisan, Paola Siviero, Federica Limongi, Marianna Noale, Stefania Maggi

**Affiliations:** ^1^Department of Medical Sciences, University of Ferrara, Ferrara, Italy; ^2^Neuroscience Institute, Aging Branch, National Research Council, Padova, Italy

**Keywords:** Mild Cognitive Impairment, Preserved Cognitive Health, dementia, cognitive status, predictors

## Abstract

Given the rising numbers of older people living with dementia, this study focuses on identifying modifiable health-related factors associated with changes in cognitive status. The predictors of 1-year conversion from Preserved Cognitive Health (PCH) and Mild Cognitive Impairment (MCI) in older adults were evaluated. Two logistic regression models were performed on data from an Italian multicenter population-based study; both included sociodemographic factors, family history of dementia (FHD), risk behaviors, and depressive symptoms. The first model considered also disease clusters, while the second one included diseases' number and biochemical parameters. The sample involved 459 participants (61.4% women, median age 75 years). Of the 80 PCH individuals at baseline, after 1 year 35 (43.8%) were stable, 44 (55.0%) progressed to MCI, none to dementia, and one to unclassified status. Of the 379 MCI participants at baseline, after 1 year 281 (74.1%) remained stable, 38 (10.0%) reverted to PCH, 15 (4.0%) progressed to dementia, and 45 (11.9%) become unclassifiable. Hypertension/bone and joint diseases cluster was the only predictor of PCH progression to MCI; age and depression were associated with MCI progression to dementia; FHD was associated with MCI reversion to PCH. More diseases and fewer white blood cells were associated with MCI progression to dementia; more diseases and lower platelets were associated with the transition from MCI to unclassifiable; higher Na and lower TSH levels were associated with MCI reversion. The treatment or management of some chronic conditions and electrolyte imbalances may help attenuate cognitive deterioration in older adults with no or MCI.

## Introduction

Dementia is one of the most burdensome conditions in older people, and its prevalence is bound to rise due to the increasing age of individuals living in both low- and high-income countries (2021 Alzheimer's disease facts and Figures, 2021). Research over the last few decades has led to identify intermediate conditions between a normal cognitive status and dementia; some interventions have been developed for these conditions in order to delay or prevent cognitive deterioration (Gauthier et al., 2006; DSM-5, American Psychiatric Association, 2013). Therefore, exploring which modifiable and unmodifiable factors are associated with improving or worsening cognitive transitions is a current priority to develop and implement preventive actions.

In this context, in addition to educational level, being involved in cognitively stimulating tasks such as physical exercise, leisure, and social activities, and having a healthy nutritional status be protective factors for cognitive decline (Mangialasche et al., 2012; Deckers et al., 2015; Kivipelto et al., 2018; Shimada et al., 2019). With regard to individuals' chronic conditions and currently-used medications, findings gathered until now on these variables have not been conclusive (Deckers et al., 2015). Some studies have shown that a higher number of chronic diseases is related to an increased risk of developing Mild Cognitive Impairment (MCI) or dementia (Vassilaki et al., 2015; Koyanagi et al., 2018; Jang and Yoon, 2019), especially in men (Koyanagi et al., 2018). Moreover, a recent work reported that even the quality of multimorbidity substantially influences dementia risk, which is increased for neuropsychiatric, cardiovascular, and sensory impairment/cancer disease patterns (Grande et al., 2021). Interestingly, according to that study, APOE ε4 and inflammatory status seemed to exacerbate the risk of dementia in association with multimorbidity (Grande et al., 2021). As regards the use of multiple drugs, a condition that often co-exists with multimorbidity, we have demonstrated that mild polypharmacy, drug–drug interactions, and anticholinergic burden have a substantial influence on the probability of progressing from MCI to dementia in older individuals (Trevisan et al., 2021).

The current study aimed to identify clinical and biochemical predictors of cognitive improvement or worsening over 1 year in older cognitively intact individuals and persons with MCI. As far as chronic conditions are concerned, we hypothesized that both their quantity and quality can affect the risk of experiencing positive or negative transitions between cognitive states. As far as biochemical parameters are concerned, we hypothesized that anemia or electrolyte imbalances would be associated with progression toward states of lower cognitive function.

## Methods

### Participants

Data came from a multicenter population-based cohort study investigating 2,337 individuals (Supplementary Figure 1) all older than 65, not demented and without serious ills, living at home or in a non-medical institution, randomly selected from the health unit lists of four Italian cities (Brescia, Schio-Vicenza, Bologna and Fermo-Ascoli Piceno) stratified by sex and age. The study, which took place between February 2002 and February 2004, was approved by the ethics committees of the local health units of the participating centers, and all the participants provided written informed consent. The population and methods of this study are fully described elsewhere (Limongi et al., 2017).

As reported in another study examining the same cohort (Limongi et al., 2017), out of the 712 initially enrolled individuals, information was available on 537 with regard to both the baseline and 1 year follow-up assessments: after 1 year 534 participated in the follow-up assessment and 3 were declared demented by a proxy respondent at that time. At each evaluation, the participants were classified into four groups based on the results of the cognitive assessments performed by neuropsychologists: Preserved Cognitive Health [PCH], Dementia, MCI, and unclassifiable.

The participants assigned to the *PCH group* were characterized by:

1) a preserved general cognitive function [age-education corrected Mini Mental State Examination, MMSE >23.8 (Folstein et al., 1975)];
2) a marginal or absent cognitive impairment [education corrected Alzheimer's Disease Assessment Scale—Cognitive subscale, ADAS-Cog score ≤17 (Fioravanti et al., 1994)];
3) a preserved memory domain [ADAS-Cog subtests: “word recall immediate,” “word recall delayed,” “word recognition immediate,” “word recognition delayed,” “remembering test instructions”; Story recall test (Spinnler and Tognoni, 1987); Rey–Osterrieth Complex Figure subtest “recall” (Caffarra et al., 2002)];
4) a preserved non-memory domain [ADAS-Cog subtest “constructional praxis” and Rey–Osterrieth Complex Figure subtest “reproduction” for praxis subdomain; ADAS-Cog subtests “commands,” “word-finding difficulty” and semantic verbal fluency test (Novelli et al., 1986) for language subdomain; phonemic verbal fluency test (Carlesimo et al., 1996) and Trail Making Test (TMT) (Amodio et al., 2002) for executive functions subdomain, TMT-B; TMT-A, TMT B-A for attention and processing speed subdomain].

All the test scores fell within 1.5 standard deviations of the mean.

The participants assigned to the *dementia group* were characterized by:

1) an impaired general cognitive function [age-education corrected MMSE ≤23.8 or an age-education Clock Drawing Test, CDT ≤5 (Sunderland et al., 1989)];
2) a deterioration in Activities of Daily Living (ADL <6) (Katz et al., 1970) or in Instrumental Activities of Daily Living (IADL <5) (Lawton and Brody, 1969);
3) a Clinical Dementia Rating Scale, CDR >0.5 (Hughes et al., 1982);
4) an impairment in at least one memory domain test as well as in at least one non-memory domain test.

The participants assigned to the MCI group were characterized by:

1) objective impairment in cognitive tests but not so severe as to fall into the dementia classification;
2) a normal general cognitive function (age-education corrected MMSE >23.8);
3) no impairment in ADL (score = 6);
4) an impairment in at least one memory or at least one non-memory domain test.

The unclassifiable group included individuals with some cognitive deficit but who did not fall into any of the other groups.

The analyses of this study focused on the 459 participants classified at the baseline as PCH (n = 80) or MCI (n = 379).

### Outcome

The study's primary outcome was the participants' cognitive progression or regression 1-year after the baseline assessment.

### Baseline Characteristics

The baseline characteristics included: age, sex, educational level, cohabitation status, prevalent working activity, family history of dementia, lifestyle (smoking and alcohol use), depressive symptoms, comorbidity, and blood sampling.

#### Educational Level

Educational level was classified as years of schooling ≤5 vs. >5 years.

#### Cohabitation Status

Cohabitation status was categorized as living alone vs. cohabitating (with spouse, conjugated sons, unmarried children, brothers/sisters, other relatives, retirement home, religious community, other).

#### Prevalent Working Activity

Prevalent working activity was dichotomized as blue-collar vs. white-collar. “Blue collar” workers consisted of unskilled/skilled craftsmen, salesman, saleswomen, farmers, and housewives whose daily work was predominantly manual. “White collar” workers consisted of officials/executives, office clerks, researchers, teachers, sales representatives, restaurant/shop managers, and other professionals whose daily work was predominantly non-manual.

#### Lifestyle Behaviors

Lifestyle behaviors were smoking habits and alcohol consumption, both dichotomized as current vs. former/never use.

#### Depressive Symptomatology

Depressive symptomatology was assessed using the Center for Epidemiologic Studies Depression Scale (CES-D Scale ≥16: score ranges from 0 to 60 points, with a higher score indicative of the presence of symptoms of depression) (Radloff, 1977).

The presence of *chronic diseases* was provided by the participants' General Practitioners. For the purposes of this study, the following were considered: hypertension, endocrinopathies, diabetes, ischemic heart disease, non-ischemic cardiopathy, cerebral vascular accidents, vasculopathy, chronic obstructive pulmonary disease (COPD), neoplasms, gastro-intestinal diseases, hepato-biliary diseases, kidney disease, bone and joint diseases, and Parkinson's disease or parkinsonisms. Cardiovascular diseases (CVD) were defined as the presence of at least one of the following: vasculopathy, ischemic heart disease, and non-ischemic cardiopathy. From the sum of these chronic conditions, we computed the total number of diseases of each individual.

#### Blood Sampling

Blood sampling after overnight fast was performed to determine the following biochemical parameters: white blood cells (× 1,000/mm^3^), red blood cells (× 1,000,000/mm^3^), hemoglobin (g/dl), hematocrit (%), Mean Globular Volume (fL), Platelets (× 1,000/mm^3^), Na (mmol/L), K (mmol/L), and TSH (MIU/L). Each parameter was evaluated with the standard reference range or dichotomized using the upper or the lower limit reference range at the local laboratory, i.e. White blood cells (normal value: 4.8–10.8 × 1,000/mm^3^); Reed blood cells (4.7–6.1 × 1,000,000/mm^3^); Hemoglobin (14–18 M. 12–16 F g/dl); Hematocrit (42–51 %); Mean corpuscular volume (80–96 fL); Platelets (150–450 × 1,000/mm^3^); Na (135–145 mmol/L); K (3.6–5.2 mmol/L); TSH (0.45–4.5 MIU/L).

### Statistical Analyses

Categorical variables are expressed as proportions and continuous variables as means and standard deviation or, if not normally distributed, as medians with interquartile ranges (IQRs). The categorical variables were compared using the χ2 test or Fisher's exact test, the continuous ones using generalized linear models after testing for homoscedasticity (Levene's test) or Wilcoxon rank-sum test, if not normally distributed. Diseases' classifications were evaluated using hierarchical cluster analysis and considering the dendrogram that was obtained. To develop the cluster analysis, the proportion of observations for which two diseases are both present was considered as the similarity measure.

Predictors of cognitive progression or regression were identified through two multivariable logistic regression models, adjusted for age, sex, and education. Models were set in order to limit the potential collinearity between the variables included and to explore the possible quantitative and qualitative influence of multimorbidity on cognitive changes. The first model (A) included cohabitation status, prevalent working activity, family history of dementia, lifestyle behaviors, depressive symptoms, and disease clusters. The second model (B) included cohabitation status, prevalent working activity, family history of dementia, lifestyle behaviors, depressive symptoms, number of diseases, and biochemical parameters (selecting in order to reduce the collinearity between them). Both models, with no missing data, were fitted using a stepwise procedure with a *p-value* for entry = 0.15 and a *p-value* for remove = 0.10. The scale of the logit for continuous variables selected in the models and all possible interactions between the variables with a *p-value* <0.10 were examined.

Odds ratios (ORs) were presented with their 95% CIs and considered statistically significant if a *p* <0.05. Statistical analyses were performed with SAS software (SAS Institute Inc, Cary, NC), version 9.4.

## Results

The characteristics of the 459 participants with PCH or MCI at baseline are shown in Table 1. The median age of the sample was 75 years and 61.4% were women. When individuals with PCH and MCI at baseline were compared, we found that the former were more likely to be younger and to have carried out white-collar activities with respect to the latter. Three disease clusters were identified (dendrogram in Supplementary Figure 2), i.e., hypertension/bone and joint diseases, gastrointestinal/CVD, and other diseases (including cerebral vascular accidents, endocrinopathies, diabetes, COPD, neoplasms, hepato-biliary diseases, kidney disease, Parkinson's disease, and Parkinsonisms).

**Table 1:** Participants' characteristics by cognitive status at baseline.

	**Missing data**	**Total**	**PCH**	**MCI**	***p-value***
		***N* = 459**	***N* = 80**	***N* = 379**	
Age years, median (IQR)		75 (71–79)	73 (69–76)	75 (71–80)	0.0003
Female sex, *n* (%)		282 (61.4)	47 (58.8)	235 (62.09)	0.5867
Years of schooling ≤5		373 (81.3)	61 (76.3)	312 (82.3)	0.2060
Living apart, *n* (%)		132 (28.8)	21 (26.3)	111 (29.3)	0.5855
Prevalent working activity, *n* (%)	2				0.0373
Blue-collar		398 (87.1)	64 (80)	334 (88.6)	
White-collar		59 (12.9)	16 (20)	43 (11.4)	
Family history of dementia, *n* (%)	33	70 (15.3)	14 (20)	56 (15.7)	0.3782
Current smoker, *n* (%)	32	44 (10.3)	10 (13.9)	34 (9.6)	0.2726
Current alcohol user, *n* (%)	30	239 (55.7)	42 (58.3)	197 (55.2)	0.6234
Diseases, *n* (%)					
Hypertension	38	261 (62.0)	45 (60)	216 (62.4)	0.6946
Endocrinopathies	40	37 (8.8)	8 (10.8)	29 (8.4)	0.5082
Diabetes	38	51 (12.1)	5 (6.9)	46 (13.2)	0.1669
Ischemic heart disease	37	70 (16.6)	10 (13.3)	60 (17.3)	0.4034
Non-ischemic cardiopathy	39	94 (22.4)	12 (16.2)	82 (23.7)	0.161
Cerebral vascular accidents	36	44 (10.4)	7 (9.3)	37 (10.6)	0.7382
Vasculopathy	37	96 (22.8)	21 (28)	75 (21.6)	0.2316
COPD disease	38	58 (13.8)	10 (13.3)	48 (13.9)	0.9022
Neoplasms	36	58 (13.7)	13 (17.3)	45 (12.9)	0.3147
Gastro-intestinal diseases	37	110 (28.4)	23 (30.7)	87 (28.0)	0.6367
Hepato-biliary diseases	37	41 (9.7)	7 (9.3)	34 (9.8)	0.9019
Kidney disease	38	30 (7.1)	6 (8)	24 (6.9)	0.7455
Bone and joint diseases	36	223 (52.7)	35 (46.7)	188 (54.0)	0.2471
Parkinson's disease or parkinsonisms	39	10 (2.4)	2 (0.7)	8 (2.3)	0.6951
Number of diseases, median (IQR)	36	3 (2–4)	2 (2–4)	3 (2–4)	0.6839
Disease clusters					
Hypertension/Bone and joint diseases, *n* (%)	37	339 (80.3)	59 (78.7)	280 (80.7)	0.6891
Gastrointestinal/CVD, *n* (%)	37	254 (60.2)	46 (61.3)	208 (59.9)	0.8234
Endocrinopathies/Diabetes/COPD/Neoplasms/Hepato- biliary diseases/Kidney disease/Parkinson's disease or parkinsonisms, *n* (%)	38	225 (53.4)	37 (49.3)	188 (54.3)	0.4311
Depressive mood, *n* (%)	6	106 (23.4)	17 (21.3)	89 (23.9)	0.6168
Blood sampling, *n* (%)					
White blood cells, × 1,000/mm^3^	44				0.5517
< LLN		79 (19.0)	12 (15.8)	67 (19.8)	
Normal (4.8–10.8)		331 (79.8)	64 (84.2)	267 (78.8)	
>ULN		5 (1.2)	0 (0.0)	5 (1.5)	
Reed blood cells, × 1,000,000/mm^3^	44				0.8312
< LLN		259 (62.4)	49 (64.5)	210 (62.0)	
Normal (4.7–6.1)		155 (37.4)	27 (35.5)	128 (37.8)	
>ULN		1 (0.2)	0 (0.0)	1 (0.2)	
Hemoglobin. g/dl	44				0.0546
< LLN		96 (23.1)	21 (27.6)	75 (22.1)	
Normal (14–18 M. 12–16 F)		316 (76.2)	53 (69.8)	263 (77.6)	
>ULN		3 (0.7)	2 (2.6)	1 (0.3)	
Hematocrit, %	44				0.2571
< LLN		297 (71.7)	57 (75)	240 (70.8)	
Normal (42–51)		116 (27.9)	18 (23.7)	98 (28.9)	
>ULN		2 (0.5)	1 (1.3)	1 (0.3)	
Mean corpuscular volume, fL	44				0.5074
< LLN		24 (5.8)	6 (7.9)	18 (5.3)	
Normal (80–96)		369 (88.9)	65 (85.5)	304 (89.7)	
>ULN		22 (5.3)	5 (6.6)	17 (5.0)	
Platelets, × 1,000/mm^3^	44				0.3189
< LLN		47 (11.3)	5 (6.6)	42 (12.4)	
Normal (150–450)		364 (87.7)	71 (93.4)	293 (86.4)	
>ULN		4 (1.0)	0 (0.0)	4 (1.2)	
Na, mmol/L	43				0.8794
< LLN		4 (0.9)	1 (1.3)	3 (0.9)	
Normal (135–145)		398 (95.7)	73 (96.1)	325 (95.6)	
>ULN		14 (3.4)	2 (2.6)	12 (3.5)	
K, mmol/L	43				0.6319
< LLN		16 (3.9)	3 (4.0)	13 (3.8)	
Normal (3.6–5.2)		397 (95.4)	72 (94.7)	325 (95.6)	
>ULN		3 (0.7)	1 (1.3)	2 (0.6)	
TSH, MIU/L	43				0.0678
< LLN		29 (7.0)	3 (4.0)	26 (7.7)	
Normal (0.45–4.5)		370 (88.9)	71 (93.4)	299 (87.9)	
>ULN		17 (4.1)	2 (2.6)	15 (4.4)	

*IQR, interquartile range; COPD, Chronic obstructive pulmonary disease; CVD, Cardiovascular diseases/Vasculopathy/Non-ischemic cardiopathy/Ischemic heart diseases; LLN, Lower Limit of Normal; ULN, Upper Limit of Normal*.

1 year later, of the 80 PCH participants, 35 (43.8%) had remained stable, 44 (55.0%) progressed to MCI, none to dementia, and one to unclassified status. Of the 379 MCI participants at baseline, after 1 year the majority remained stable (*n* = 281, 74.1%), 38 (10.0%) reverted to PCH, 15 (4.0%) progressed to dementia, and 45 (11.9%) become unclassifiable.

When the PCH participants who remained stable were compared with those who progressed to MCI, we found that the latter were more likely to present conditions in hypertension/bone and joint diseases cluster (Table 2).

**Table 2:** Participants' characteristics by cognitive progression or reversion.

	**From PCH**		**From MCI**			
	**Stable PCH**	**Progression to MCI**	**Stable MCI**	**Progression to dementia**	**Progression to unclassified**	**Reversion to PCH**
	***N* = 35**	***N* = 44**	***N* = 281**	***N* = 15**	***N* = 45**	***N* = 38**
Age years, median (IQR)	71 (68–74)	73 (70–76.5)	75 (71–79)	**80.0 (78.0**–86.0)^*^	**77.0 (71.0**–91.0)^*^	75 (70–79)
Female sex	19 (54.3)	27 (61.4)	171 (60.9)	7 (46.7)	33 (73.3)	24 (63.2)
Years of schooling ≤5	25 (71.4)	35 (79.6)	233 (82.9)	10 (66.7)	36 (80.0)	33 (86.8)
Living apart	7 (20.0)	14 (31.8)	81 (28.8)	4 (26.7)	13 (28.9)	13 (34.2)
Blue–collar	26 (74.3)	37 (84.1)	249 (89.3)	13 (86.7)	39 (86.7)	33 (86.8)
Family history of dementia	5 (17.2)	9 (22.5)	5 (17.2)	3 (21.4)	4 (10.0)	9 (22.5)^*^
Current smoker	7 (23.3)	3 (7.3)	24 (9.1)	0 (0.0)	6 (15.0)	4 (11.1)
Current alcohol user	19 (63.3)	23 (56.1)	156 (58.9)	6 (40.0)	16 (39.0)^*^	19 (52.8)
Depressive mood	6 (17.1)	10 (22.7)	63 (22.6)	6 (40.0)	12 (28.6)	8 (21.6)
Disease clusters						
Hypertension/Bone and joint diseases	22 (66.7)	36 (87.8)^*^	208 (80.9)	13 (92.9)	33 (80.5)	26 (74.3)
Gastrointestinal/CVD diseases	19 (57.6)	26 (63.4)	148 (57.8)	9 (60.0)	32 (78.1)^*^	19 (54.3)
Other	18 (54.6)	18 (43.9)	135 (52.5)	7 (46.7)	25 (62.5)	21 (61.8)
Number of diseases, median (IQR)	2 (1–4)	3 (2–4)	2 (2–4)	**3 (2**–5)^*^	**3 (3**–4)^*^	3 (2–4)
White blood cells < LLN × 1,000/mm^3^	6 (17.7)	6 (14.6)	52 (20.6)	**6 (42.9)**	4 (10.8)	5 (13.9)
White blood cells > ULN × 1,000/mm^3^	0 (0.0)	0 (0.0)	4 (1.6)	0 (0.0)	0 (0.0)	1 (2.8)
Reed blood cells < LLN × 1,000,000/mm^3^	22 (64.7)	27 (65.9)	155 (64.5)	9 (64.3)	23 (62.2)	23 (63.9)
Reed blood cells > ULN × 1,000,000/mm^3^	0 (0.0)	0 (0.0)	1 (0.4)	0 (0.0)	0 (0.0)	0 (0.0)
Hemoglobin < LLN g/dl	12 (35.3)	9 (22.0)	51 (20.2)	5 (35.7)	9 (24.3)	10 (27.8)
Hemoglobin >ULN g/dl	0 (0.0)	2 (4.9)	1 (0.4)	0 (0.0)	0 (0.0)	0 (0.0)
Hematocrit < LLN %	28 (82.4)	28 (68.3)	178 (70.6)	10 (71.4)	29 (78.4)	23 (63.9)
Hematocrit > ULN %	0 (0.0)	1 (2.4)	1 (0.4)	0 (0.0)	0 (0.0)	0 (0.0)
Mean corpuscular volume < LLN fL	2 (5.9)	3 (7.3)	15 (6.0)	0 (0.0)	2 (5.4)	1 (2.8)
Mean corpuscular volume > ULN fL	3 (8.8)	2 (4.9)	12 (4.8)	0 (0.0)	2 (5.4)	3 (8.3)
Platelets < LLN × 1,000/mm^3^	4 (11.8)	1 (2.4)	26 (10.3)	**4 (28.6)**	8 (21.6)^*^	4 (11.1)
Platelets > ULN × 1,000/mm^3^	0 (0.0)	0 (0.0)	4 (1.6)	0 (0.0)	0 (0.0)	0 (0.0)
Na < LLN mmol/L	0 (0.0)	1 (2.4)	2 (0.8)	1 (7.1)	0 (0.0)	0 (0.0)
Na > ULN mmol/L	1 (2.9)	1 (2.4)	7 (2.8)	0 (0.0)	0 (0.0)	5 (13.9)^*^
K < LLN mmol/L	1 (2.9)	2 (4.9)	10 (4.0)	1 (7.1)	1 (2.6)	1 (2.9)
K > ULN	1 (2.9)	0 (0.0)	2 (0.8)	0 (0.0)	0 (0.0)	0 (0.0)
TSH < LLN MIU/L	2 (5.9)	1 (2.4)	17 (6.8)	1 (7.1)	2 (5.3)	6 (16.7)^*^
TSH > ULN	0 (0.0)	2 (4.9)	12 (4.8)	0 (0.0)	1 (2.6)	2 (5.6)

*IQR, interquartile range; CVD, Cardiovascular diseases/Vasculopathy/Non-ischemic cardiopathy/Ischemic heart diseases; COPD, Chronic obstructive pulmonary disease; LLN, Lower Limit of Normal; ULN, Upper Limit of Normal. The disease cluster “Other” includes cerebral vascular accidents, endocrinopathies, COPD, neoplasms, hepato-biliary diseases, kidney disease, Parkinson's disease or parkinsonisms*.

* *p < 0.05 and p ≤ 0.06 derived by comparing participants experiencing progression or reversion transitions, with those stable in the baseline cognitive status*.

*Values in bold indicate statistically significant results*.

The individuals who progressed from MCI to dementia had a significantly higher number of diseases and tended to have lower values of white blood cells and platelets than the stable MCI participants.

Those who progressed from MCI to unclassifiable status were more likely to be older and to present a higher number of diseases, especially gastrointestinal/CVD and lower platelet values. They also showed a lower percentage of current alcohol consumption.

Participants who reverted from MCI to PCH more frequently had a family history of dementia, higher Na, and lower TSH levels.

### Predictors of Cognitive Progression or Regression

The results of the multivariable logistic regression models A and B, adjusted for age, sex, and schooling years, are reported in Table 3.

**Table 3:** Predictors of cognitive progression or reversion from preserved cognitive health or mild cognitive impairment.

	**PCH progression to MCI**			**MCI progression to dementia**			**MCI progression to unclassified**			**MCI reversion to PCH**		
**Model A**	***N*** **=** **64 (37 vs. 27)**			***N*** **=** **250 (13 vs. 237)**			***N*** **=** **269 (32 vs. 237)**			***N*** **=** **268 (31 vs. 237)**		
	***p-value***	**OR**	**CI 95%**	***p-value***	**OR**	**CI 95%**	***p-value***	**OR**	**CI 95%**	***p-value***	**OR**	**CI 95%**
Age years	0.248	1.1	0.95–1.21	**0.005**	**1.2**	**1.05**–**1.27**	0.056	1.1	1.00–1.12	0.300	1.0	0.91–1.03
Female sex	0.464	1.5	0.49–4.88	0.275	2.1	0.56–7.71	0.129	0.5	0.22–1.22	0.276	0.8	0.36–1.77
Years of schooling ≤ 5	0.256	2.0	0.61–6.56	0.073	0.3	0.09–1.12	0.356	0.7	0.25–1.63	0.863	1.1	0.39–3.08
Living apart												
Blue-collar (vs white collar)												
Family history of dementia										**0.029**	**2.6**	**1.11**–**6.28**
Current smoker												
Current alcohol user												
Depressive mood				0.060	3.4	0.95–12.02						
Disease clusters												
Hypertension/Bone and joint diseases	**0.041**	**4.0**	**1.06**–**14.93**									
GI/CVD diseases												
Other												
**Model B**	***N*** **=** **61 (35 vs. 26)**			***N*** **=** **227 (13 vs. 214)**			***N*** **=** **239 (25 vs. 214)**			***N*** **=** **245 (31 vs. 214)**		
	***p-value***	**OR**	**CI 95%**	***p-value***	**OR**	**CI 95%**	***p-value***	**OR**	**CI 95%**	***p-value***	**OR**	**CI 95%**
Age years	0.167	1.1	0.97–1.22	**0.006**	**1.2**	**1.05**–**1.32**	**0.027**	**1.1**	**1.01**–**1.17**	0.783	1.0	0.91–1.04
Female sex	0.840	1.1	0.36–3.46	0.203	2.3	0.63–8.65	0.360	0.6	0.24–1.69	0.432	0.7	0.29–1.51
Years of schooling ≤5	0.241	2.0	0.62–6.53	0.139	0.4	0.10–1.38	0.610	0.8	0.25–2.24	0.482	1.2	0.41–3.33
Living apart												
Blue-collar (vs white collar)												
Family history of dementia										0.079	2.3	0.91–5.92
Current smoker												
Current alcohol user												
Depressive mood												
Number of diseases				**0.021**	**1.5**	**1.07**–**2.19**	**0.027**	**1.5**	**1.14**–**1.88**			
White blood cells < LLN (4.8 ×1,000/mm^3^)				**0.033**	**4.2**	**1.13**–**15.33**						
Hemoglobin < LLN (14 M, 12 F g/dl)												
Mean corpuscular volume > ULN (96 fL)												
Platelets < LLN (150 ×1,000/mm^3^)							**0.015**	**3.8**	**1.30**–**10.88**			
Na > ULN (145 mmol/L)										**0.035**	**7.3**	**1.92**–**27.76**
K < LLN (3.6 mmol/L)												
TSH < LLN (0.45 MIU/L)										**0.024**	**3.6**	**1.19**–**11.05**

*OR, Odds Ratio; CI, Confidence Intervals; CVD, Cardiovascular diseases/Vasculopathy/Non-ischemic cardiopathy/Ischemic heart diseases; COPD, Chronic obstructive pulmonary disease; GI, gastrointestinal; LLN, Lower Limit of Normal; ULN, Upper Limit of Normal; the disease cluster “Other” includes cerebral vascular accidents, endocrinopathies, COPD, neoplasms, hepato-biliary diseases, kidney disease, Parkinson's disease or parkinsonisms*.

*Values in bold indicate statistically significant results*.

In Model A, the disease cluster of hypertension/bone and joint diseases was the only factor independently associated with PCH progression to MCI. Advancing age and, marginally, depressive mood were associated with the progression from MCI to dementia. Reversion from MCI to PCH was associated with a family history of dementia.

In Model B, the risk of progression from MCI to dementia increased with advancing age, a higher number of diseases, and lower white blood cells. Older age, a higher number of diseases, and lower platelet values were associated with the transition from MCI to unclassifiable. Some predictors of progression from MCI to unclassified were the same as those regarding the progression from MCI to dementia. This can be explained by the fact that the MCIs that become unclassifiable were characterized by a worsening in general cognitive status, activities of daily living, and cognitive tests/functions (Supplementary Figure 3). Finally, the presence of Na levels >145 mmol/L and TSH <0.45 MIU/L were associated with an increased chance of reverting from MCI to PCH.

## Discussion

The current study demonstrates that some modifiable, or at least manageable clinical factors were associated with the progression or reversion of states of normal cognition or MCI in community-dwelling older adults. In particular, the disease cluster characterized by hypertension and osteoarticular diseases seemed to accelerate the development of MCI from PCH. Instead, the accumulation of chronic diseases, the presence of depressive mood, and lower leukocytes values seemed to increase the risk of MCI transition to dementia, while higher serum sodium and lower TSH levels were associated with the regression from MCI to normal cognition.

As far as changes in cognitive status over a year's time, we found that around half of the PCH participants in our sample progressed to MCI, while almost 44% remained cognitively intact. As concerns MCI, the rate of progression to dementia was 4%, while 10% reverted to PCH, and 12% became unclassifiable at the follow-up assessment. This picture is in line with findings published previously (Di Carlo et al., 2007; Ganguli et al., 2011). Indeed, the estimates for MCI reversion rates ranged from 8% in clinical-based studies to 25% in population-based ones (Canevelli et al., 2016), while annual rates were ~5–10% for MCI progression to dementia (Mitchell and Shiri-Feshki, 2009).

The analysis of the clinical factors associated with the transition from PCH to MCI showed that the cluster composed of hypertension and osteoarticular diseases was linked to a higher risk of progression to MCI. Hypertension is a vascular risk factor that has already been associated with MCI development, especially with the non-anamnestic type (Reitz et al., 2007), and inversely related to the risk of reverting from MCI to normal cognition (Xue et al., 2019). The mechanisms by which hypertension could cause neurodegeneration include the development of subcortical white matter lesions, alterations in the blood-brain barrier, and oxidative stress (Reitz et al., 2007). These factors make MCI a common finding among individuals with hypertension, affecting approximately one out of three patients (Qin et al., 2021). Interestingly, along with hypertension, osteoarticular diseases also belong to the cluster at higher risk of MCI development. The chronic inflammation underlying common bone and joint disorders such as osteoarthritis, as well as the related chronic pain and limitations in functional status and physical activity are likely the main determinants of the detrimental effect of these diseases on cognitive performance (Koyanagi et al., 2018; Weber et al., 2019). Moreover, the finding by a recent study that osteoarthritis was associated with a steeper decline in total gray matter volumes over time in individuals free from dementia (Wu et al., 2021) further supports our results.

With regard to the individuals with MCI at baseline, we found that the probability of progression to dementia was directly associated with a higher number of diseases. The impact of multimorbidity on neurodegeneration has been previously underlined and may be linked to states of chronic inflammation and oxidative stress exacerbated by the accumulation of chronic diseases (Vassilaki et al., 2015; Koyanagi et al., 2018; Jang and Yoon, 2019). Interestingly, in our sample, the presence of depressive mood but no specific disease clusters were associated with that transition. This is only partially in line with a recent work that reported that neuropsychiatric, cardiovascular, and sensory impairment/cancer disease patterns had a substantial impact on dementia risk (Grande et al., 2021). On the other hand, we also found an association between lower white blood count and MCI progression. This result appears to be at odds with the infectious hypothesis for Alzheimer's disease, which theorizes that a pathogenic agent contributes to the development of an inflammatory status promoting neurodegeneration (Seaks and Wilcock, 2020). However, further investigations on the inflammatory patterns that characterize certain types of dementia have uncovered alterations both in leukocyte numbers and composition. In Alzheimer's disease, for example, along with higher values of monocytes and neutrophils, some researchers found low lymphocyte and basophil levels (Shad et al., 2013; Chen et al., 2017). As we have no information on the participants' leukocyte formula, we are unable to formulate any conclusion on this point, and future studies will need to examine possible associations.

Besides older age and a higher number of diseases, lower platelet values increased the probability of transitions from MCI to the unclassifiable group, which consisted of individuals reporting a worsening of the general cognitive status, the activities of daily living and cognitive tests/functions, but who did not fall under the other categories. Studies currently available in the literature do not consistently support the association between platelets and cognitive function in older people, although activated platelets seem to play a substantial role in the pathogenesis of certain types of dementia, such as Alzheimer's disease (Catricala et al., 2012; Socha et al., 2019) and vascular dementia (Ahn et al., 2002).

Finally, the probability of reversion from MCI to PCH in our sample was directly associated with a family history of dementia, higher serum sodium, and lower TSH levels. As regards the family history of dementia, our result is not in line with the current literature, but it is likely related to low statistical power since the number of participants with MCI and family history of dementia in our sample was quite small. Concerning sodium and TSH levels, our study corroborates previous findings. Indeed, hyponatremia was consistently associated with an increased risk of dementia in several observational studies and the mechanisms mediating the effect may include the chronic activation of the renin-angiotensin system, inflammation, oxidative stress, and mitochondria and hippocampal dysfunctions (Renneboog et al., 2006; Cooper et al., 2015; Xu et al., 2015; Chung et al., 2017). As regards thyroid hormones, a recent large cohort study found that hypothyroidism was associated with an increased risk of dementia and that the relationship was influenced by age, comorbidities, and the duration of elevated TSH (Thvilum et al., 2021). These results support previous studies finding that both hypo- and hyperthyroidism were associated with a higher dementia risk (Davis et al., 2003; Annerbo and Lökk, 2013; Elbadawy et al., 2020), while subclinical thyroid dysfunction with mild TSH alteration did not seem to influence cognitive decline (Rieben et al., 2016). In our population, lower TSH values emerged as a protective factor for MCI reversion, but the unavailability of records regarding FT3 and FT4 levels did not allow us to distinguish the presence of subclinical hyper- and hypothyroid dysfunctions. Further research on the thyroid-brain axis is needed given that cardiovascular risk factors may be associated with both thyroid and cognitive disorders and the thyroid may affect oxidative stress, cholinergic activity, and the amyloid-β protein precursor expression (Annerbo and Lökk, 2013).

The strengths of this study lie in its prospective and multicenter design and the evaluation of several sociodemographic and health-related variables, which may provide an overview of the multidimensionality of dementia pathogenesis. Its limitations are linked to the fact that we did not consider subjective cognitive complaints to detect MCI cases since preliminary results showed that a substantial number of participants would have been unclassifiable. Moreover, the short follow-up and the fact that information regarding variables used for classifying the participants was missing (probably due to their worsening cognitive status) did not allow us to detect a substantial number of incident dementia cases, limiting the statistical power of our analyses. Studies with longer observation periods are certainly more appropriate to detect the predictors of dementia development, which usually occurs over longer timeframes. Nonetheless, such a short follow-up allowed us to assess the transitions across intermediate cognitive states, which could be evident over smaller time intervals (Larrieu et al., 2002; Farias et al., 2009; Petersen, 2011; Sachdev et al., 2013; Davis et al., 2018; Ashraf-Ganjouei et al., 2021; Thaipisuttikul et al., 2022). Finally, we have no data on the type of dementia diagnosed at the follow-up evaluation. In conclusion, this study confirms that modifiable or manageable clinical factors are associated with short-term cognitive deterioration or improvements in older adults with no cognitive deficits or MCI. Interventions aiming to manage chronic conditions, delay the accumulation of diseases, and treat electrolyte and thyroid imbalances may help to hamper the progression of cognitive deterioration in advanced age.

## Data Availability Statement

The datasets analyzed during the current study are available from the corresponding author on reasonable request. Requests to access these datasets should be directed to PS, paola.siviero@in.cnr.it.

## Ethics Statement

The study was approved by the Ethics Committees of the Local Health Units of the Participating Centers. The patients/participants provided their written informed consent to participate in this study.

## Author Contributions

SM and CT conceptualized the study. PS performed the statistical analysis. CT and PS wrote the original draft. CT, PS, FL, MN, and SM edited and revised critically. All authors contributed and approved the final version.

## Funding

This study was supported by a Special Program of the Ministry of Health (Special program ex art. 12, comma 2, lett B D.Lvo n. 502/92).

## Conflict of Interest

The authors declare that the research was conducted in the absence of any commercial or financial relationships that could be construed as a potential conflict of interest.

## Publisher's Note

All claims expressed in this article are solely those of the authors and do not necessarily represent those of their affiliated organizations, or those of the publisher, the editors and the reviewers. Any product that may be evaluated in this article, or claim that may be made by its manufacturer, is not guaranteed or endorsed by the publisher.
