# Supplementary material for: Health-Related Predictors of Changes in Cognitive Status in Community-Dwelling Older Individuals
Source: Front Aging Neurosci. 2022 Jun 20;14:876359. doi: 10.3389/fnagi.2022.876359 (PMC9252613; doi:10.3389/fnagi.2022.876359)
Supplement: Supplementary file 1 [file Data_Sheet_1.PDF]

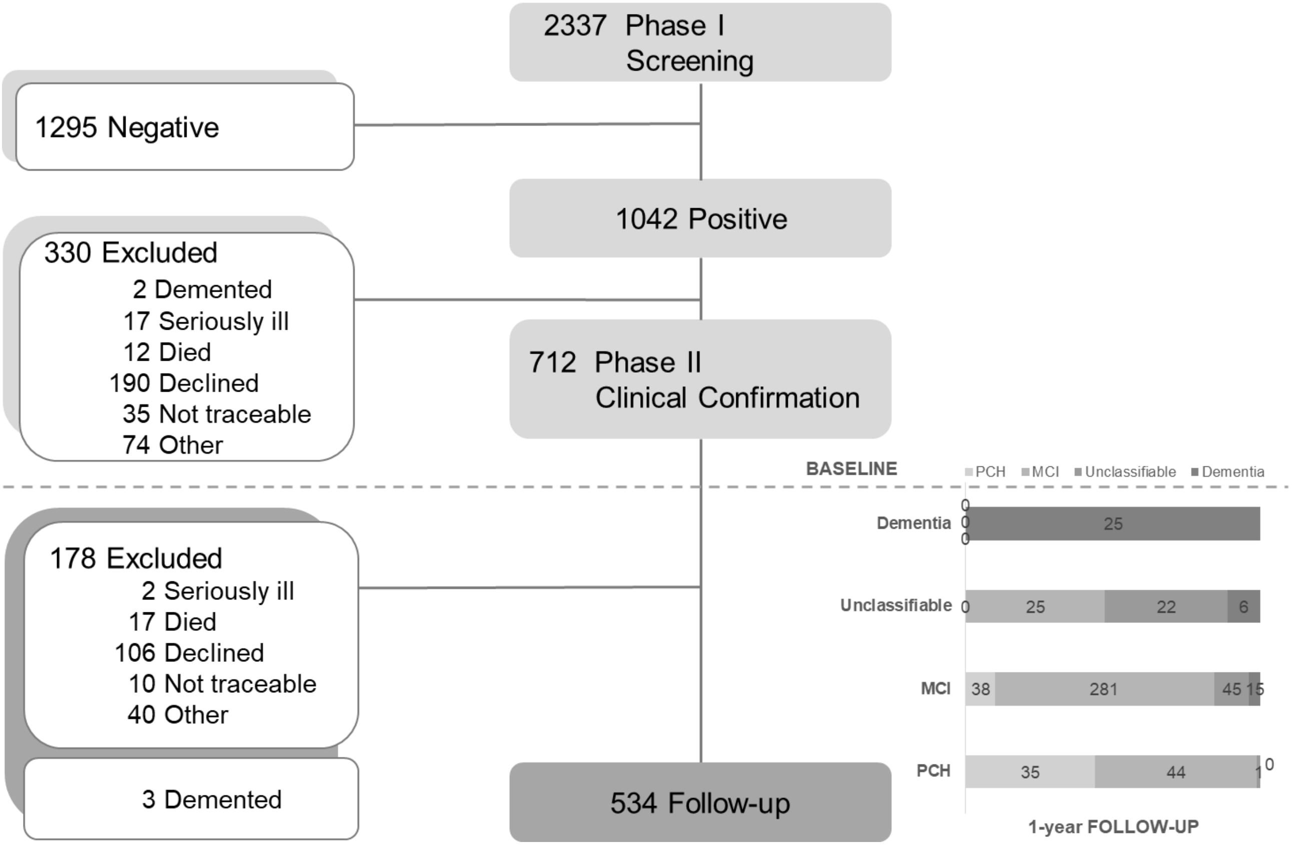

Supplementary Figure 1: Study flow-chart

Abbreviations: PCH, Preserved Cognitive Health; MCI, Mild Cognitive Impairment.

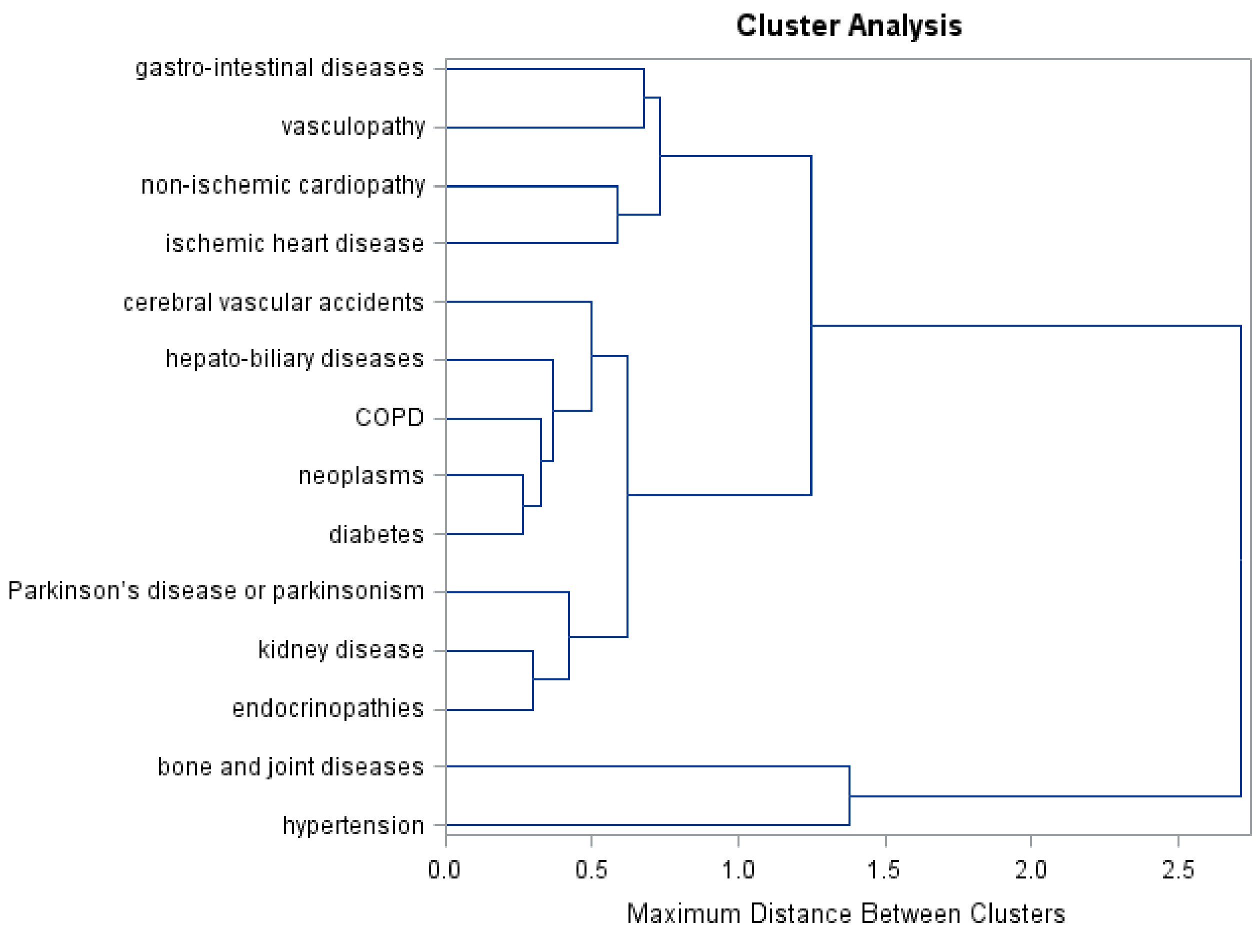

Supplementary Figure 2. Dendrogram for diseases of the hierarchical clustering.
Abbreviations: COPD: chronic obstructive pulmonary disease.

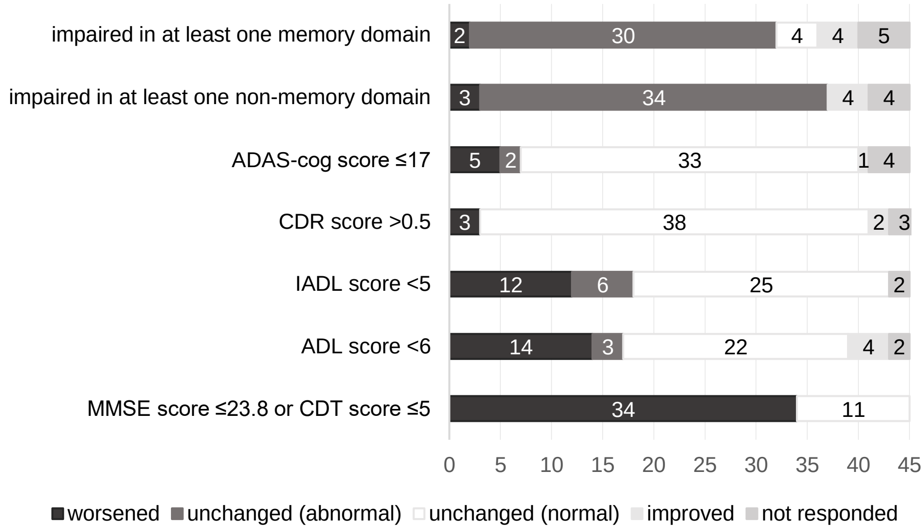

Supplementary Figure 3. Progression from MCI to unclassified according to the classification criteria.
Abbreviations: ADAS-Cog: Alzheimer's Disease Assessment Scale - Cognitive subscale; CDR: Clinical Dementia Rating Scale; IADL: Instrumental Activities of Daily Living; ADL: Activities of Daily Living; MMSE: Mini Mental State Examination; CDT: Clock Drawing Test; MCI: Mild Cognitive Impairment.
